# SIAH1 reverses chemoresistance in epithelial ovarian cancer via ubiquitination of YBX-1

**DOI:** 10.1038/s41389-022-00387-6

**Published:** 2022-03-10

**Authors:** Wujiang Gao, Lu Chen, Li Lin, Meiling Yang, Taoqiong Li, Hong Wei, Chunli Sha, Jie Xing, Mengxue Zhang, Shijie Zhao, Qi Chen, Wenlin Xu, Yuefeng Li, Xiaolan Zhu

**Affiliations:** 1 Reproductive Center, The Fourth Affiliated Hospital of Jiangsu University, Zhenjiang, China; 2 Department of Central Laboratory, The Fourth Affiliated Hospital of Jiangsu University, Zhenjiang, China; 3 The first people’s hospital of Nantong, Nantong, China; 4 Department of Radiology, The Affiliated Hospital of Jiangsu University, Zhenjiang, China

**Keywords:** Ovarian cancer, Ubiquitylation

## Abstract

Chemoresistance is a severe outcome among patients with epithelial ovarian cancer (EOC) that leads to a poor prognosis. YBX-1 has been shown to cause treatment failure and cancer progression in EOC. However, strategies that directly target YBX-1 are not yet conceivable. Here, we identified that SIAH1 which was downregulated in chemoresistant EOC samples and cell lines functioned as novel E3 ligases to trigger degradation of YBX-1 at cytoplasm by RING finger domain. Mechanistic studies show that YBX-1 was ubiquitinated by SIAH1 at lys304 that lead to the instability of its target m5C-modified mRNAs, thus sensitized EOC cells to cDDP. Overexpression of SIAH1 enhanced the antitumor efficacy of cisplatin in vitro and in vivo, which were partially impaired by ectopic expression of YBX-1 or depletion of YBX-1 ubiquitination. In summary, our data identify the SIAH1/YBX-1 interaction as a therapeutic target for overcoming EOC chemoresistance.

## Introduction

Epithelial ovarian cancer (EOC) accounts for ~90% of all ovarian cancers which is the most fatal of all gynecological malignancies, mostly diagnosed at advanced stages with poor prognosis [1–4]. As a representative of platinum anticancer drugs, cisplatin (DDP) plays a critical role in the treatment of EOC in clinical chemotherapy [5, 6]. However, a significant proportion of patients who are initially highly responsive to DDP treatment experience a relapse within 2 years and fail to respond to existing treatments due to acquired resistance [7, 8]. Therefore, revealing the mechanisms of DDP resistance and exploring new therapeutic targets are the keys to prolonging the survival of patients.

**Fig. 1:**
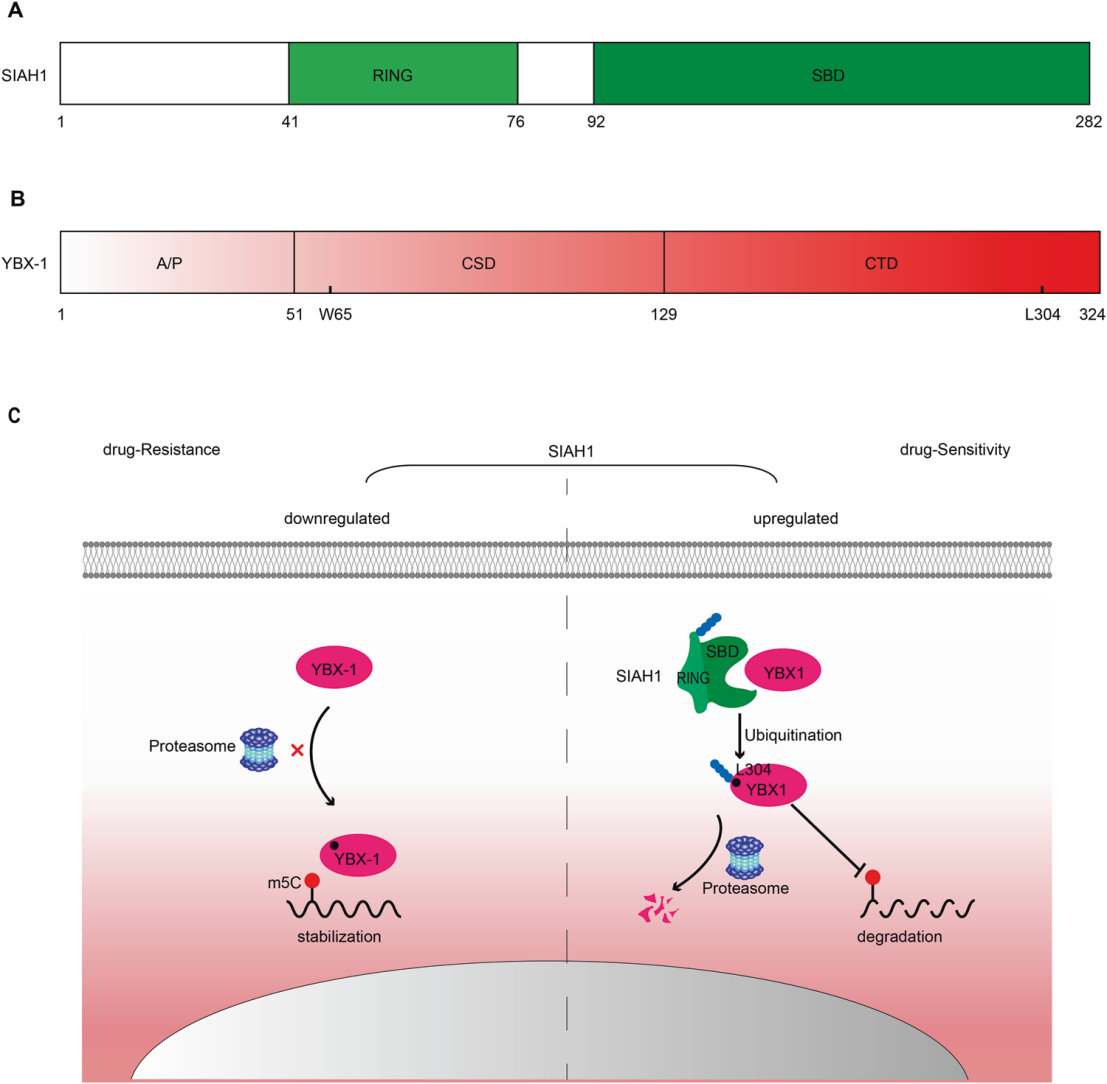
The secondary structure of proteins and a diagrammatic sketch of the protective effects of SIAH1 in EOC by reversing drug resistance. **A** Schematic of the structure of the SIAH1 protein. The relative positions of the catalytic RING domain and a substrate-binding domain cold shock domain (CSD). **B** Domain organization of YBX-1 proteins. Relative positions of the amino acid residues CTD are shown. AP-domain: alanine/proline-rich domain, CDS: cold shock domain, and Lysine 304 ubiquitination site is indicated throughout the 324AA long protein structure. **C** Model: SIAH1, promoting YBX-1 ubiquitylation and proteasome degradation to regulate the stability of the mRNA E2F5, YY1, and RCC2, ultimately leading to a platinum-sensitive EOC phenotype.

The maintenance of protein homeostasis plays a critical role in many biological processes including tumorigenesis, which is closely related to the post-translational modification of proteins, by covalent attachment of ubiquitin [9–12]. Studies have shown that ubiquitination is closely related to chemotherapy resistance [13–15]. The ubiquitination machinery is structured in a hierarchical cascade in which ubiquitin-activating enzyme (E1), ubiquitin-conjugating enzyme (E2), and ubiquitin ligase (E3) act sequentially to promote the transfer of Ub onto substrates [11, 16–19]. The diverse family of E3s with specific E2s determines the exquisite specificity of proteins fated for ubiquitination [20, 21]. The seven in absentia homologs (SIAHs) comprise a family of highly conserved E3 ubiquitin ligases with a catalytic RING domain, two zinc finger domains, and a substrate-binding domain [11, 22–25]. SIAH1 is a human member of the highly conserved SIAH family [11, 26, 27] (Fig. 1A). An increasing number of SIAH1 protein substrates have been authenticated, and they are mainly related to fundamental cellular processes, including hypoxia response pathways (AKAP1, HIPK2, FIH, and PHDs), DNA damage response (TIN2, TRF2, and HIPK2), and cancer inhibition (ACK1, PEG10, DX2, and TRAF4) [28–32].

Emerging a growing number of studies have shifted the focus to the effect of SIAH1 on cancers recently [30, 32, 33]. For example, SIAH1 exerts inhibitory effects on the progression and development of many kinds of malignant tumors, including colorectal cancer, hepatocellular carcinogenesis, breast cancer, and prostate cancer [29, 33, 34], and the ubiquitin ligase activity of SIAH1 largely contributes to its tumor-inhibiting effect. Data from The Cancer Genome Atlas (TCGA) show that SIAH1 gene downregulation is a common genetic alteration in different kinds of cancers. Consistently, SIAH1 is reported to be significantly downregulated in various cancer types, including drug-resistant ovarian cancer [35]. In addition, SIAH1 has been reported to modulate drug resistance mediated by multidrug resistance 1 (MDR1)/P-glycoprotein in the cancer cells [35, 36].

Human Y box-binding protein (YBX-1) was identified as a DNA and RNA-binding protein participating in various processes, such as DNA repair, mRNA splicing, RNA stabilization, translational repression, and transcription regulation [37–42]. It is a member of the highly conserved cold shock domain (CSD) family of proteins, and consists of three recognized domains: an amino-terminal region; a central cold shock domain (CSD), which is responsible for its specific interaction with nucleic acids and contains RNP-1 motif; and a C-terminal domain (CTD), which may be responsible for protein–protein interactions [41, 43–45] (Fig. 1B). Furthermore, YBX-1 has been reported to promote the stabilization of m5C-modified mRNAs [46] and overexpression of YBX-1 is involved in the drug resistance of several human tumors [47, 48]. YBX-1 promotes the pathogenesis of human urothelial carcinoma through stabilizing elevated levels of m5C modification mRNA HDGF [46]. In EOC, silencing YBX-1 significantly reduces the drug resistance of EOC cells [49–51]. Because of the oncogenic role and promoting drug resistance functions of YBX-1, it is conceivable that the ubiquitin enzymes destabilizing YBX-1 could contribute to cancer treatment

In this study, we aimed to explore the function and underlying molecular mechanism of SIAH1 in regulating drug resistance in EOC and to investigate its target genes and their downstream effects, so as to provide a novel target for the prevention and reversal of drug resistance in EOC.

## Results

### SIAH1 is downregulated in ovarian cancer and inhibits ovarian cancer proliferation, invasion, migration, and drug resistance in vitro

**Fig. 2:**
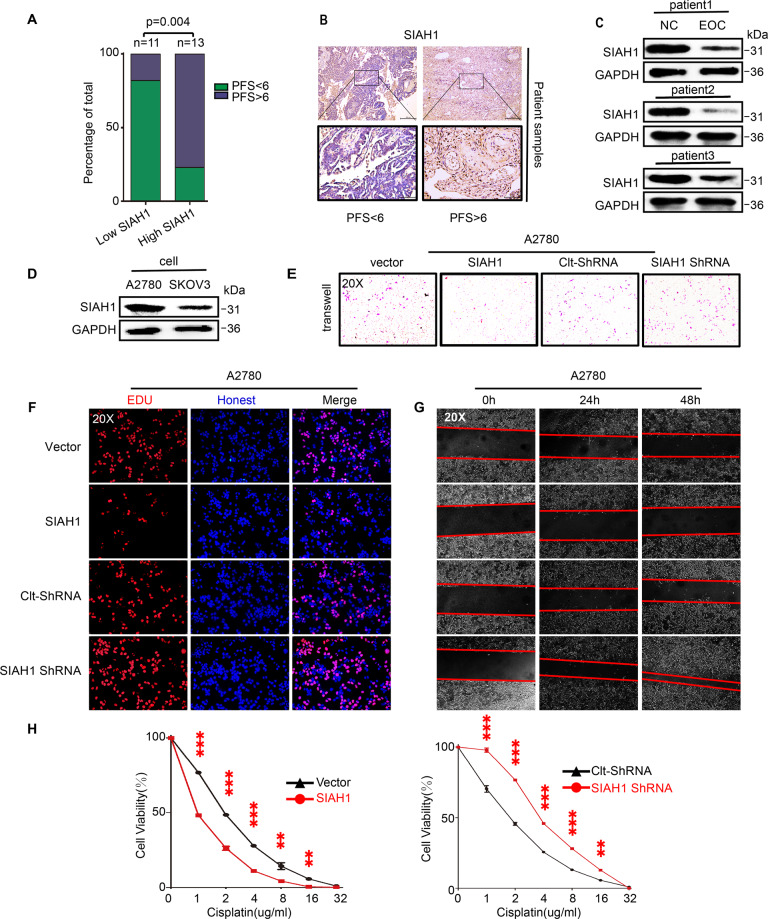
SIAH1 inhibits the proliferation, invasion, migration, and drug resistance of EOC cells. **A** A significant association was found between SIAH1 expression with PFS of EOC (SIAH1 low = SIAH1 staining positive rate <25%, SIAH1 high = SIAH1 staining positive rate > =25%) in 24 patients. **B** IHC of SIAH1 expression in EOC tissues of patients who were platinum-sensitive (PFS >6) and -resistant (PFS <6). **C** SIAH1 was downregulated in the serum of EOC patients (EOC) compared with healthy controls (HC). **D** The protein level of SIAH1 was downregulated in SKOV3 cells compared with A2780 cells. **E** Transwell invasion assay, **F** EdU assay, **G** wound-healing assay, and **H** CCK-8 assay was performed to detect the cell invasion, proliferation, migration, and viability of A2780 cells separately.

To investigate the role of SIAH1 in EOC and obtain initial understanding of SIAH1 expression patterns, we first retrieved and analyzed the data from the online database GEPIA. It was shown that decreased SIAH1 mRNA existed in part of the human malignant tumors compared with normal tissues, including EOC (Supplementary Fig S1A), and high SIAH1 expression was significantly related to the improvement of progression-free survival (PFS) in EOC patients (the patients were divided into two groups according to their SIAH1 IHC staining positive rate) (Fig. 2A and Supplementary Fig. S1B). Then, we focused on EOC and found that SIAH1 was remarkably decreased in tumor tissue derived from platinum-resistant (PFS < 6) patients compared with platinum-sensitive (PFS > 6) patients (Fig. 2B and Supplementary Fig. S1C), as well as in EOC patients serum compared with healthy controls (HC) (Fig. 2C). Moreover, SIAH1 protein level was downregulated in the platinum-resistant cell line (SKOV3) compared with the platinum-sensitive cell line (A2780) (Fig. 2D).

Next, the effects of SIAH1 on the chemoresistance of EOC were assessed. As shown in (Fig. 2E–H and Supplementary Fig. S1D–F), SIAH1 overexpression resulted in a distinct decrease in the ability of invasion, proliferation, migration, and drug resistance of SKOV3 and A2780 cells. Conversely, knockdown of SIAH1 significantly enhanced the proliferation, migration, invasion, and drug resistance (Fig. 2E–H and Supplementary Fig. S1D–F), indicating that SIAH1 restrains the invasion, proliferation, migration, and drug resistance of EOC cells.

### SIAH1 expression renders DDP sensitization in vivo

**Fig. 3:**
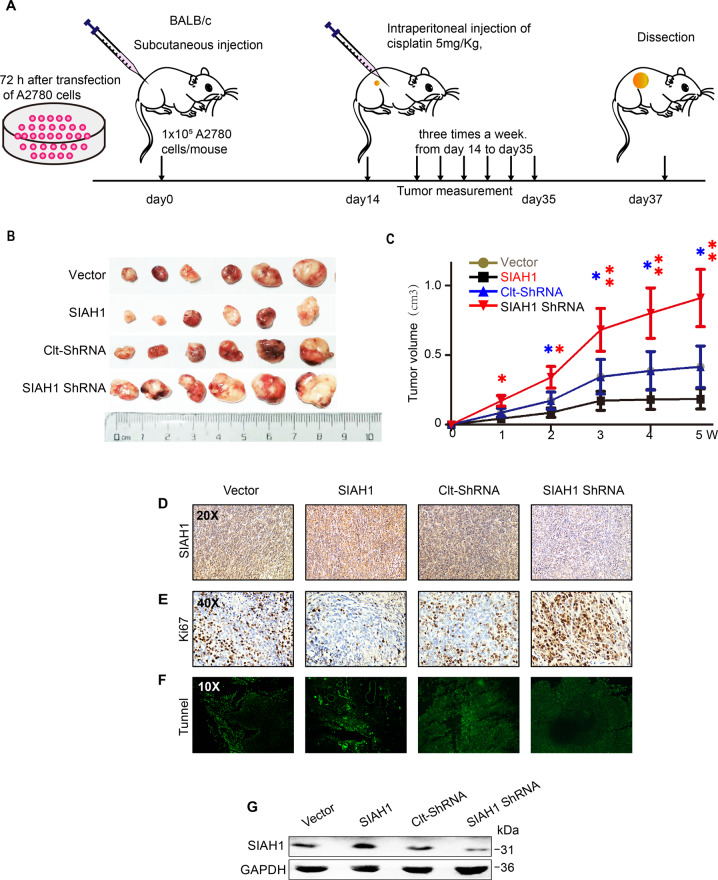
SIAH1 expression renders DDP sensitization in vivo. **A** Lv-SIAH1, Lv-Vector, or Lv-shSIAH1 transfected A2780 cells were subcutaneously injected into BALB/c nude mice, DDP (5 mg/kg) was injected into the center of the xenograft tumors twice per week for 3 consecutive weeks. **B** Representative images of tumors in xenografts in each group. **C** The tumor growth curve was shown. **D**, **E** Representative image of SIAH1 and KI-67 staining in tumors treated as indicated above. **F** Representative photographs of TUNEL analysis in tumors treated as indicated above. **G** WB for SIAH1 in tumor tissues collected from mice in each group (tissues from six tumors in the same group were mixed into one sample).

To further evaluate the role of SIAH1 in vivo, the xenograft tumor models were established through injecting A2780 ovarian cancer cells infected with Lv-SIAH1 or Lv-shSIAH1 into the subcutaneous tissue of nude mice. Five weeks later, we sacrificed the mice and measured the tumor volume (Fig. 3A). It was observed that tumors from mice injected with Lv-SIAH1-infected A2780 cells were significantly smaller than those from mice processed with control cells, while tumors from mice injected with Lv-shSIAH1 were visibly larger (Fig. 3B). We calculated the diameter of subcutaneous tumors volume and observed that SIAH1 overexpression decreased the drug resistance of EOC cells in vivo (Fig. 3C). In addition, the high level of SIAH1 was associated with the low proliferative capacity of the tumors (Fig. 3D, E, G and Supplementary Fig. S2A, B). Furthermore, as shown in (Fig. 3F and Supplementary Fig. S2C), TUNEL assay confirmed the attenuation of apoptotic effect following knockdown of SIAH1. These data correspond with our in vitro findings, supporting a tumor-inhibiting effect of SIAH1 on EOC.

### SIAH1 interacts with and modulates YBX-1

**Fig. 4:**
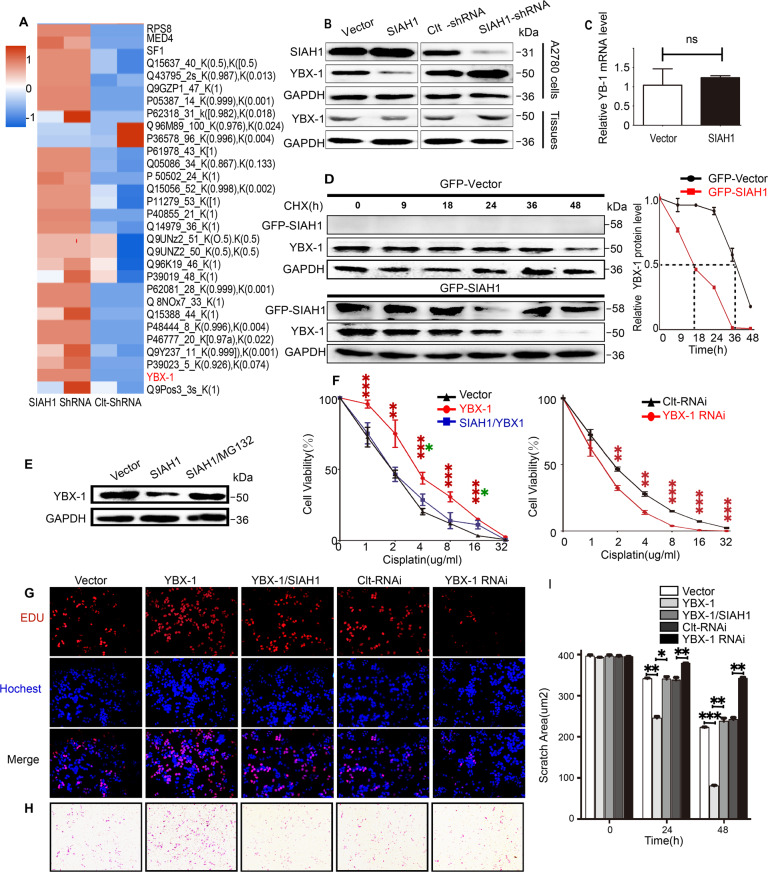
SIAH1 reduces the stability of YB-1 protein. **A** Protein mass spectrometry was used to analyze the expression levels of related proteins in cells after the knockout of SIAH1. **B** Relative expression levels of YBX-1 proteins after SIAH1 overexpression and knockout. (up, samples were derived from A2780 cells; down, samples were derived from mouse xenograft tumors tissue). **C** Relative YBX-1 mRNA expression in SIAH1-overexpression cells. **D** HEK293T cells were transfected with GFP-Vector and GFP-SIAH1 and treated with cycloheximide (CHX) for the indicated times. The bar graph shows quantified protein levels of YBX-1. **E** HEK293T cells were transfected with Vector and SIAH1, respectively, and the third group was treated with MG132 for 6 h. The protein levels of YBX-1 were detected by immunoblot. **F** CCK-8 assay, **G** EdU assay, **H** Transwell invasion assay, **I** wound-healing assay were applied to analyze the cell viability proliferation, invasion, and migration of A2780 cells separately.

Currently, a comprehensive set of all SIAH1 substrates is not available. To explore the potential proteins that interact with SIAH1, we performed liquid chromatography-tandem mass spectrometry (LC-MS/MS) for ubiquitinated proteins in HEK293T cells overexpressing or knocking down SIAH1 (Fig. 4A). The potential interacting proteins for SIAH1 were determined based on the following criteria: (1) unique peptides of the ubiquitinated proteins were more than 2; and (2) the difference in ubiquitination modification of the protein peptide was greater than 2 compared with the control group. Among them, YBX-1 was identified from the above LC-MS/MS analysis (Supplementary Table S1). Therefore, YBX-1 is a potential novel SIAH1-binding protein.

Considering the role of YBX-1 in promoting chemoresistance, we explored whether YBX-1 is involved in SIAH1-mediated inhibition of EOC chemoresistance. We overexpressed and knocked down SIAH1 in A2780 cells, and the expression of YBX-1 was analyzed by WB. The results show that the YBX-1 protein levels were negatively regulated by SIAH1 in cells, as well as in xenograft tumors overexpressing or knocking down SIAH1 mentioned above (Fig. 4B). As a proof-of-concept experiment, we examined the YBX-1 mRNA level in control and SIAH1-overexpressing HEK293T cells. As shown (Fig. 4C), the relative mRNA level of YBX-1 was not influenced by SIAH1 overexpression, suggesting that the regulation of YBX-1 by SIAH1 does not occur through transcription. Combined with the result of LC-MS/MS analysis, we hypothesized that one mechanism by which SIAH1 may downregulate YBX-1 is ubiquitination-promoted degradation. To test this hypothesis, we first examined the half-life of YBX-1 by treating control and SIAH1-overexpressing HEK293T cells with cells protein synthesis inhibitor cycloheximide (CHX). As shown (Fig. 4D), CHX treatment generated a dramatic decrease of YBX-1 protein level over the time course, leading to a shorter half-life of YBX-1 in SIAH1-overexpressing cells as compared to Control cells. Overall, our data suggest that SIAH1 decreases YBX-1 protein stability. To confirm that YBX-1 protein degradation occurs through the ubiquitin–proteasome pathway, HEK293T cells overexpressing SIAH1 were incubated with the proteasome inhibitor MG132. Interestingly, this analysis indicated that exposure to MG132 upregulated YBX-1 expression in SIAH1-overexpressing HEK293T cells, as shown in (Fig. 4E). Therefore, SIAH1 regulates YBX-1 stability through the proteasomal degradation pathway.

In view of the above findings, we next assessed the role of YBX-1 in EOC cells. As shown in (Fig. 4F–I and Supplementary Fig. S3A–C), the YBX-1 overexpression-induced promotion of cell drug resistance, proliferation, invasion, and migration, which could be largely reversed by overexpression of SIAH1 in A2780 cells. Suggesting that SIAH1 inhibits EOC drug resistance, proliferation, migration, and invasion via promoting YBX-1 degradation in EOC cells.

### SIAH1 directly promotes YBX-1 ubiquitination

**Fig. 5:**
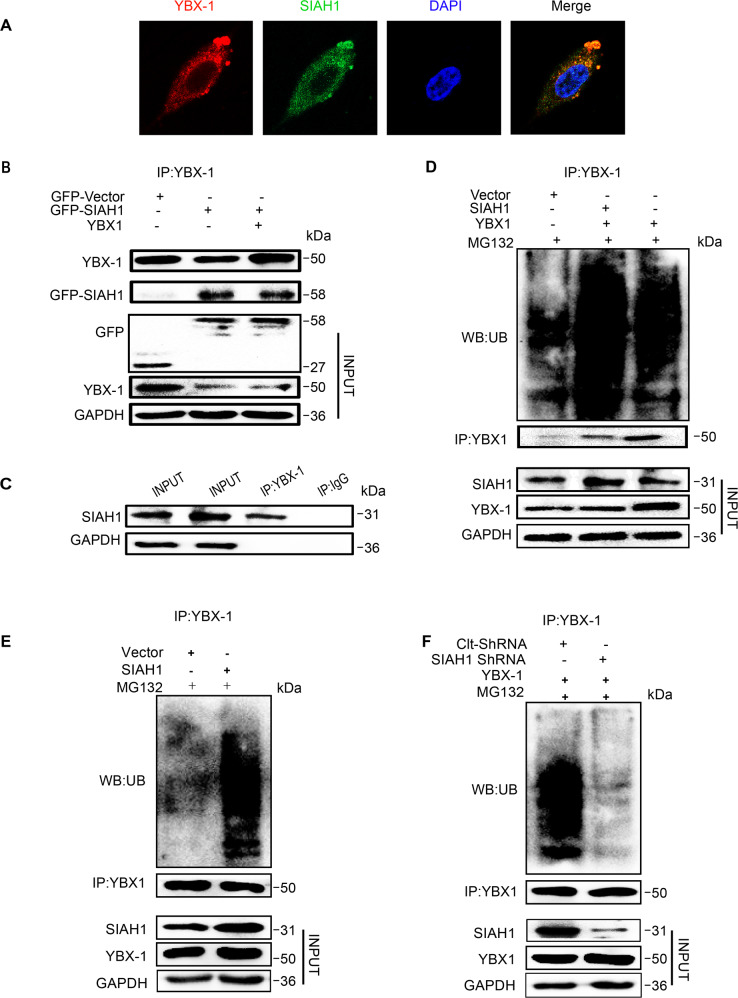
YBX-1 is ubiquitinated by E3 ligase SIAH1. **A** A2780 cells were cotransfected with GFP-SIAH1 plasmids (green) and Cherry-YBX-1 plasmids (red). **B** IP of GFP-SIAH1 and YBX-1. 293 cells stably expressing GFP-SIAH1 were pulled down by anti-YBX-1 and immunoblotted with anti-GFP. **C** IP of endogenous SIAH1 and YBX-1. 293 cells lysates were pulled down by anti-YBX-1 and anti-IgG, then immunoblotted with anti-SIAH1. **D**, **E** HEK293T cells were cotransfected with GFP-SIAH1 and FLAG-YB-1 or GFP-SIAH1 Individually, The cell lysates were immunoprecipitated with anti-YBX-1 antibody. The ubiquitinated YBX-1 was detected by immunoblotting using an anti-UB antibody. Cells were treated with 10 μM MG132 for 6 h before pulling down. **F** HEK293T cells were cotransfected with YBX-1 and SIAHI ShRNA, YBX-1 and Clt-ShRNA, respectively. The cell lysates were immunoprecipitated with anti-YBX-1 antibody. The ubiquitination was checked the same as mentioned above.

To further explore the mechanism of SIAH1-induced proteasomal degradation of YBX-1, a double-transfection study was performed to confirm whether SIAH1 colocalized with YBX-1. GFP-SIAH1 was concomitantly expressed with Cherry-YBX-1 by transient transfection of A2780 cells, and then co-cultured with MG132 after 36 h. The colocalization of cytoplasmic SIAH1 and YBX-1 in ovarian cancer cells was detected by confocal microscopy (Fig. 5A), supporting the direct interaction between SIAH1 and YBX-1 proteins. Moreover, applying mutual co-IP assays, the interaction of exogenous SIAH1 with YBX-1 was sustained in HEK293T cells transfected with GFP-tagged SIAH1 (Fig. 5B). However, in the control group, which expressed GFP-Vector fusion proteins, GFP protein could not be detected in the immunoprecipitates WB pulled down by anti-YBX-1 antibody. Furthermore, we confirmed that YBX-1 could be coimmunoprecipitated by endogenous SIAH1 but not IgG in HEK293T cells (Fig. 5C).

SIAH1 has been verified to specifically mediate the degradation of its substrate proteins via the ubiquitin–proteasome pathway [52]. Accordingly, the present study investigated whether SIAH1 facilitates YBX-1 degradation by promoting YBX-1 ubiquitination. HEK293T cells were cotransfected with SIAH1 and YBX-1 plasmids and subsequently cultured with MG132. The ubiquitination of YBX-1 in cell lysates was revealed by immunoprecipitation with anti-YBX-1 antibody and WB with anti-UB antibody. In cells overexpressing YBX-1, ubiquitin-conjugated YBX-1 proteins were detected in the anti-UB immunoprecipitates, suggesting that YBX-1 was slightly ubiquitinated. In cells coexpressing SIAH1 and YBX-1, ubiquitin-conjugation of YBX-1 was markedly increased (Fig. 5D). In addition, the ubiquitination of endogenous YBX-1 was promoted by ectopic expression of SIAH1 (Fig. 5E). To further explore whether SIAH1 regulates YBX-1 ubiquitination, we knocked down SIAH1 using shRNA. The depletion of SIAH1 in HEK293T cells largely downregulated YBX-1 ubiquitination (Fig. 5F), suggesting that SIAH1 ubiquitinates YBX-1 directly.

### SIAH1 ubiquitinated YBX-1 at its K304 through the RING domain

**Fig. 6:**
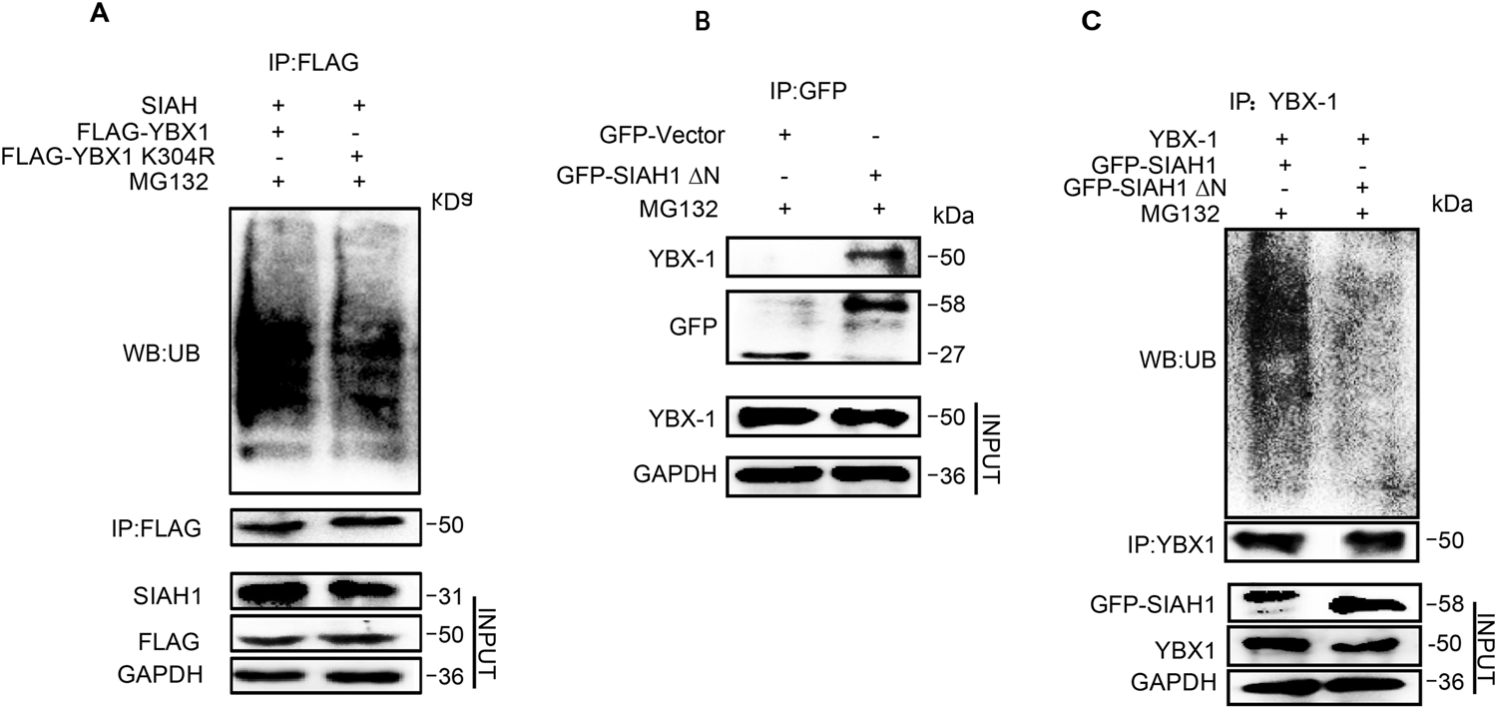
SIAH1 ubiquitinated YBX-1 at its K304 through RING domain. **A** HEK293T cells were transfected with the indicated constructs, and treated with MG132 for 6 h. Then the cell lysates were immunoprecipitated with anti-FLAG antibody. Detcting ubiquitinated YBX-1 with anti-UB antibody. **B** IP of GFP-SIAH1∆N and YBX-1. 293 cells stably expressing GFP-SIAH1∆N were pulled down by anti-YBX-1 and immunoblotted with anti-GFP. **C** HEK293T cells were cotransfected with YBX-1 and GFP-SIAH1, YBX-1 and GFP-SIAH1∆N, respectively. The levels of Ubiquitinated YBX-1 were detected by immunoblot.

Next, we attempted to determine the K residue(s) on YBX-1 where SIAH1-linked ubiquitin takes place. According to the results of ubiquitinated proteins LC-MS/MS analysis mentioned above, YBX-1 is possibly ubiquitinated at K304 lysine residues (Supplementary Table S2). To verify this, we mutated the K304 lysine residue of YBX-1 to arginine to generate YBX-1 K304R mutant. As shown in (Fig. 6A), the ubiquitination levels of the YBX-1 K304R mutant were significantly decreased compared to wild-type YBX-1, suggesting that K304 may be the predominant ubiquitination site in vivo. Previous studies have shown that the canonical E3 RING motif SIAH1 gene encoding at its C-terminus end, which is necessary for its E3 ligase function [53] (Supplementary Fig. S8B). In order to better comprehend the biological function of the SIAH1 RING domain in mediating YBX-1 ubiquitination degradation, we constructed encoding N-terminal truncated form of SIAH1 (GFP-SIAH1∆N). First, coimmunoprecipitation was performed in HEK293T cells transfected with expression vectors for GFP-SIAH1, GFP-SIAH1∆N, and YBX-1 to explore whether SIAH1∆N could still bind to YBX-1. As shown in (Fig. 6B), the deletion of the RING domain had no effect on SIAH1 binding to YBX-1. To test the effect of its deletion on YBX-1 ubiquitination, HEK293T cells were transfected with expression plasmids for GFP-SIAH1, GFP-SIAH1∆N, and YBX-1 and subsequently cultured with MG132. Cell lysates were collected and immunoprecipitated with an anti-YBX-1 antibody, followed by immunoblotting with an anti-UB antibody to estimate the ubiquitination of YBX-1. SIAH1 overexpression promoted YBX-1 ubiquitination, but the SIAH1 RING domain deletion mutant devoid of E3 ligase activity failed to trigger this ubiquitination event despite its intact binding affinity to YBX-1 (Fig. 6C). Given the above, these results are consistent with the conception that the RING finger domain of E3 is necessary for YBX-1 ubiquitination that leads to degradation.

### SIAH1-mediated YBX-1 ubiquitination sensitizes EOC to cDDP in vivo

**Fig. 7:**
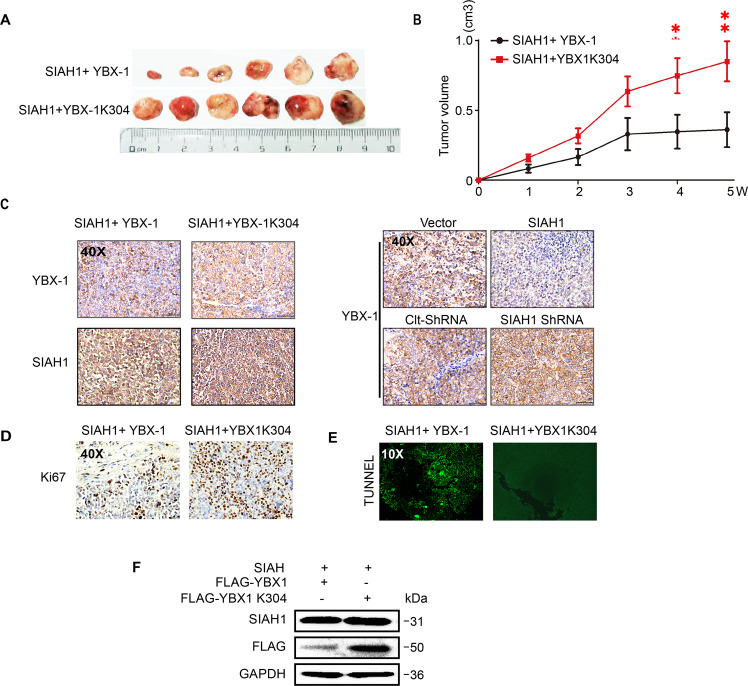
SIAH1-mediated YB-1 ubiquitination sensitizes EOC to cDDP in vivo. **A** YBX-1 knocked down A2780 cells were transfected with the indicated constructs subcutaneously injected into BALB/c nude mice. The xenograft tumor experiment were performed same as mentioned above. Representative images of tumors in xenografts in each group (**B**) corresponding growth curve, (**C**) IHC of YBX-1 and SIAH1 expression in tumor tissues of each group. **D** Representative images of KI-67 staining in tumors treated. **E** Representative photographs of TUNEL analysis in tumors treated. **F** WB for YBX-1 and SIAH1 in tumor tissues collected from mice in each group (tissues from six tumors in the same group were mixed into one sample).

To further explore whether SIAH1 also confers DDP sensitivity via YBX-1 ubiquitin degradation in vivo, we transfected Lv-SIAH1 together with the Lv-YBX-1 or Lv-YBX-1 K304R mutant into YBX-1 stable knockdown A2780 cells which were constructed with LV-YBX-1-shRNA in advance, and subcutaneously injected them into BALB/c nude mice. As shown in (Fig. 7A, B), the tumors from the mice injected with A2780 cells harboring Lv-SIAH1 and Lv-YBX-1 were significantly smaller and more sensitive to DDP compared with mice injected with A2780 cells harboring Lv-SIAH1 and Lv-YBX-1 K304R after YBX-1 knockdown. These results suggest that YBX-1 K304 is a determinant site for inhibiting EOC cell growth in vivo, which is mediated by SIAH1.

IHC staining and WB (Fig. 7C, F) were then performed to assess the SIAH1 and YBX-1 expression levels in xenograft tumors. The results are consistent with our in vitro findings, showing a strong negative correlation between SIAH1 and YBX-1 in tumor tissues. Meanwhile, a high level of SIAH1 was correlated with lower YBX-1 in the tumors from the mice injected with A2780 cells harboring Lv-SIAH1 and Lv-YBX-1, but not in the tumors from the mice processed with A2780 cells harboring Lv-SIAH1 and Lv-YBX-1 K304R, further confirming that SIAH1 is a YBX-1 ubiquitinase that could destabilize YBX-1 by ubiquitinating it at K304 lysine residues. Furthermore, enhanced Ki-67 expression in YBX-1 K304R-overexpressing tumors (Fig. 7D) was detected. Conversely, TUNEL assay (Fig. 7E) indicated an apparent increase in the number of TUNEL-positive cells in the tumors of mice injected with cells harboring Lv-YBX-1 when compared with cells harboring Lv-YBX-1 K304R. Collectively, these results suggest that SIAH1 contributes to the DDP sensitivity of A2780 cells in vivo by mediating YBX-1 ubiquitination at K304.

### SIAH1 indirectly regulates m5C-modified RNA stability through ubiquitinating YBX-1

Considering SIAH1 serves as a tumor-inhibiting role in EOC by mediating the ubiquitination and degradation of YBX-1, which acts as a DNA and RNA-binding protein that specifically recognizes mRNA transcripts modified by C5-methylcytosine (m5C), promotes the stability of these transcripts, and thus regulates tumor progression. Therefore, we hypothesized that SIAH1 indirectly regulates mRNA stability by destabilizing YBX-1.

**Fig. 8:**
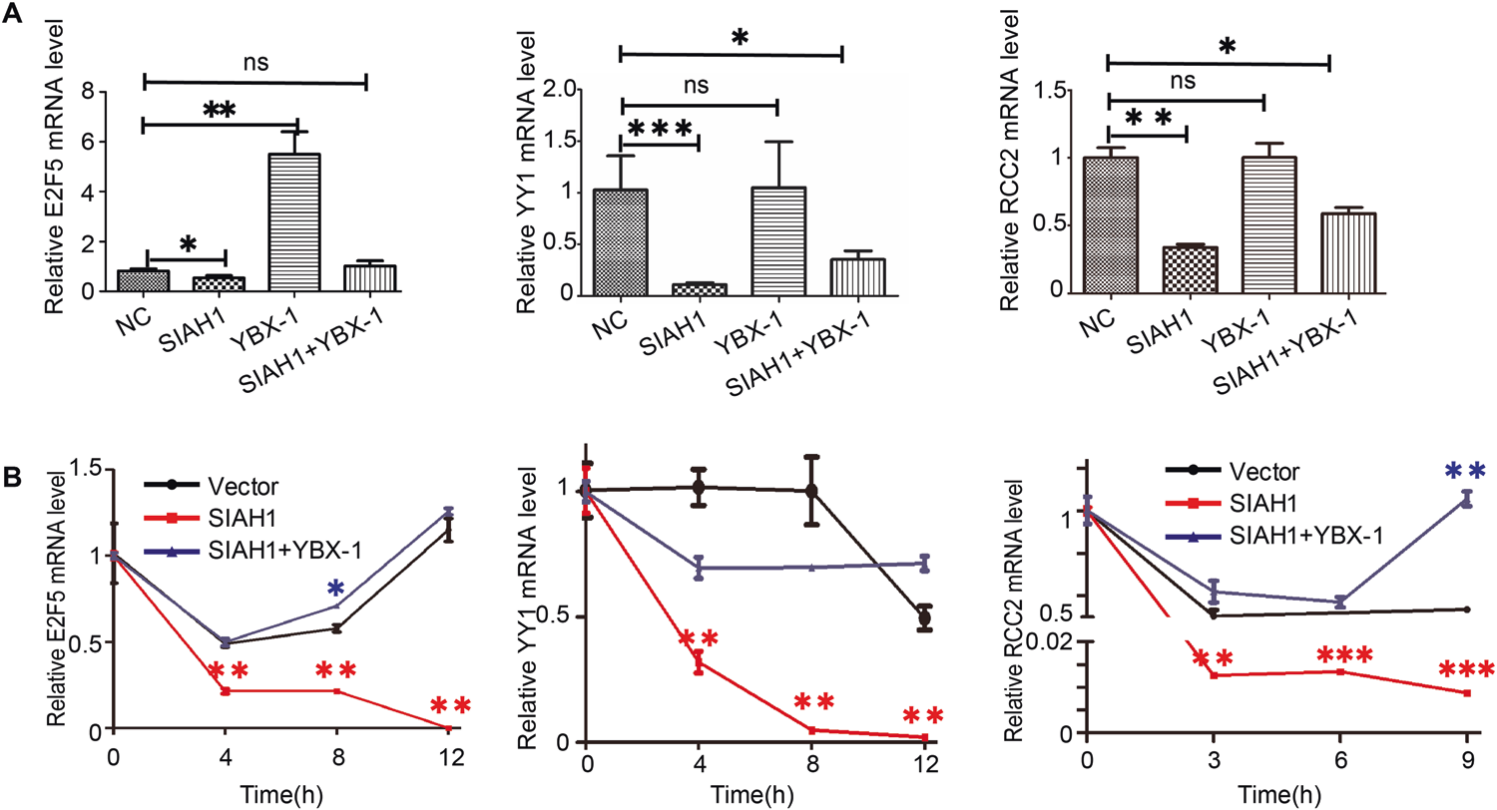
SIAH1 indirectly regulates RNA stability through ubiquitinating YBX-1. **A** A2780 cells were transfected with Vector, SIAH1, YBX-1, and cotransfected with SIAH1 and YBX-1, respectively. qRT-PCR detected relative mRNA expression. **B** qRT-PCR detected the mRNA E2F5, YY1, and RCC2 expression of A2780 cells were transfected with Vector, SIAH1, and cotransfected with SIAH1 and YBX-1, respectively.

In view of the above circumstance, we identified 51 genes with m5C sites and then combined them with YBX-1 Ribo-Seq and RIP-Seq results in HEK293T cells [46, 54], selecting 39 genes that interact with YBX-1. Further analysis of the 39 genes revealed that 13 genes were related to drug resistance of EOC, and subsequent experimental analysis was carried out. To prove our hypothesis, we first detected the relative mRNA level in A2780 cells transfected with SIAH1 and YBX-1 separately. Substantial increases in the expression level of E2F5, YY1, and RCC2 mRNA were observed in A2780 cells transfected with YBX-1. In contrast, overexpression of SIAH1 significantly downregulated the expression of these mRNAs. Specifically, this effect could be significantly restored by overexpressing YBX-1 (Fig. 8A). This result is consistent with our conjecture, indicating that SIAH1 could regulate mRNA expression through ubiquitination of YBX-1.

Next, to further explore whether the regulation of mRNA expression by SIAH1 is achieved by reducing their stability. We treated A2780 cells overexpressing vector or SIAH1 and coexpressing SIAH1 with YBX-1 with actinomycin D as indicated, collecting the cells at each specified time to determine the levels of mRNA expression. As shown in (Fig. 8B), compared with the control cells, the degradation of mRNA in cells transfected with SIAH1 was significantly accelerated. And the effect caused by SIAH1 overexpression could be significantly reversed by coexpressing YBX-1. These results provide support for the YBX-1-dependent roles of SIAH1 in regulating m5C-modified mRNA stability. Overall, these results indicate that a SIAH1–YBX-1-E2F5/YY1/RCC2 axis exists and that this axis plays an essential role in regulating the DDP sensitivity and tumor suppressor function of SIAH1 (Fig. 1C).

## Discussion

Cisplatin resistance to chemotherapy is a major challenge to the efficacy of EOC treatment and is associated with a poor clinical prognosis [5]. However, little is known about the latent molecular mechanisms of drug resistance. A clearer understanding of the molecules involved in these processes and their regulation would therefore help in designing therapeutic strategies to surmount chemoresistance and prevent relapses.

The pivotal and unique regulatory mechanism that controls the overall protein levels of eukaryotic cells and is associated with a multitude of cellular and molecular processes, such as cell cycle control, DNA damage response, nuclear translocation, endocytosis, post-Golgi trafficking, and innate immunity, is the central theme of ubiquitination and ubiquitin–proteasome system (UPS) [55, 56]. It has gradually become a potential topic of interest to the biopharmaceutical industry. SIAH family proteins are known to have proven E3 ubiquitin ligase activities and involved in a multitude of physiological events and pathophysiological conditions, such as in tumors. SIAH1 earmarks protein substrates for ubiquitination and destruction by the proteasome. In addition, researchers have shown that the expression of SIAH1 is often downregulated or absent in multiple human cancer cell types, including hepatocellular carcinoma, colorectal cancer, breast cancer, lung cancer and bladder cancer [57–59], and plays an important regulatory role in sensitizing multidrug-resistant cells to chemotherapeutic agents [35]. In concordance with these reports, our study found that SIAH1 was downregulated in EOC patients and tumor tissue derived from DDP-sensitive EOC patients compared with DDP-resistant EOC patients. Functional studies revealed that ectopic overexpression of SIAH1 alleviated DDP resistance and suppressed invasion, proliferation, and tumor growth of EOC cells both in vitro and in vivo, and exogenous knockdown of SIAH1 generated the opposite effects. These findings suggest that SIAH1 may play a tumor suppressor role and may be a potential prognostic marker of EOC.

To further explore the underlying mechanism by which SIAH1 regulates the drug sensitivity of EOC, we employed ubiquitinated protein LC-MS/MS analysis in EOC cells and found that YBX-1 was a novel substrate of SIAH1 which was enriched in EOC and involved in tumorigenesis and drug resistance. In addition, previous studies have shown that the YBX-1 protein could be degraded by RBBP6 through the RING finger domain via the ubiquitin–proteasomal pathway, which also illustrates that the YBX-1 has a lysine site that can be ubiquitinated [60]. Consistently, our study showed that SIAH1 overexpression decreased YBX-1 expression and inhibited the ability of YBX-1 to promote proliferation, invasion, tumor growth, and DDP resistance in EOC cells, thus enhancing the chemosensitivity of EOC cells.

Furthermore, a double-transfection study confirmed that SIAH1 colocalized with cytoplasmic YBX-1. Using a protein pull-down assay, we ascertained in the present work that the YBX-1 protein is a SIAH1-binding protein, and SIAH1 overexpression causes YBX-1 degradation by the ubiquitin–proteasome pathway. Based on the results of ubiquitinated peptides LC-MS/MS analysis, we further confirmed the major ubiquitination site of YBX-1 at lysine 304. In addition, SIAH1 theoretically coupled specific protein substrates through substrate-binding domain (SBD) and induced substrate ubiquitination and degradation through N-terminal RING domain [53], which is also consistent with our experimental results: SIAH1 but not SIAH1∆N mediated degradation of the YBX-1 protein via the ubiquitin–proteasome pathway, but SIAH1∆N could still bind to YBX-1 in the pull-down assays. These results further verified that the RING domain of SIAH1 is necessary for the E3 ligase function of SIAH1 to ubiquitinate YBX-1.

YBX-1, as a DNA- and RNA-binding protein, is a major protein bound to mRNAs in mammalian cells and related to many RNA-dependent events [61]. For example, it regulates the transcription of many genes, including those implicated in proliferation and differentiation. YBX-1 has also been involved in packaging mRNAs into messenger ribonucleoprotein particles (mRNPs), alternative mRNA splicing, and recombination of DNA, and it regulates the stability and translational activity of mRNA [46, 61]. It has been reported that YBX-1 can bind to m5C-containing mRNAs and recruit the ELAVL1 protein, which acts as a mRNA stability maintainer to prevent mRNA decay [46]. Herein, we verified the stability of three genes (E2F5, YY1, and RCC2) [62–64] that could interact with and stabilized by YBX-1 and possessed m5C sites were indirectly downregulated by SIAH1. Although the downstream pathway of the SIAH1-YBX-1-E2F5/YY1/RCC2 axis requires further investigation, our results suggest that SIAH1-mediated YBX-1 ubiquitination, which leads to accelerated degradation of these mRNAs, may play an important role in chemosensitization.

Altogether, our results suggest that SIAH1 regulates EOC cell drug sensitivity by mediating degradation of the YBX-1 protein through the ubiquitin–proteasome pathway and revealed the SIAH1–YBX-1-E2F5/YY1/RCC2 axis as a promising therapeutic target.

## Conclusions

Our study suggests that SIAH1, as an E3 ubiquitin ligase, is a low expression in EOC and might be a previously unrecognized new target for EOC chemotherapy. Dysregulation of SIAH1 expression plays a central role in stimulating the chemoresistance and progression of EOC, partially through promoting YBX-1 ubiquitylation and degradation to modulate the stability of the mRNAs encoding E2F5/YY1/RCC2.

## Materials and methods

### Cell lines and cell culture

In this study, we used the EOC cell lines SKOV3 and A2780 from Central Laboratory of Zhenjiang Fourth People’s Hospital. HEK293T cells were donated by the Central Laboratory of Jiangsu University. RPMI 1640 (HyClone) with 10% fetal bovine serum (FBS) were used in cells culture. DDP was provided by the Pharmacy Department of the Fourth Affiliated Hospital of Jiangsu University. The Cycloheximide (CHX) and proteasome inhibitor MG132 were purchased from (MedChemExoress, shanghai, China). All cells were maintained at 37 °C in 5% CO_2_.

### Patient tissue and serum samples

patient tissue and serum samples were obtained from patients after receiving informed consent in The First Affiliated Hospital of Jiangsu University and the Fourth Affiliated Hospital of Jiangsu University. Each group had 12 patient samples.

### Cell transfection

Cells were incubated in six-well plates the day before transfection, and then transfected with plasmids or shRNAs when the cells have reached 70% fusion as indicated in the figure legends using Lipofectamine2000 reagent following the manufacturer’s protocol (#11668019, Invitrogen, Carlsbad, CA, USA). Harvesting cells at 42–48 h post transfection for future experiments.

### Plasmids and lentivirus

The Flag-tagged YBX-1-expressing plasmid, Cherry-tagged YBX-1-expressing plasmid, Flag-tagged YBX-1 K304R-expressing plasmid (Point mutant), Flag-tagged YBX-1-RNAi plasmid, The GFP-tagged SIAH1∆N-expressing plasmid (knock out the RING domain of SIAH1), The GFP-tagged SIAH1-expressing plasmid, GFP-tagged SIAH1-RNAi plasmid, and SIAH1-expressing plasmid were purchased from Genechem Co., Ltd (Shanghai, China). Lentivirus:LV-SIAH1, LV-SIAH1-RNAi, LV-YBX-1, LV-YBX-1 K304R, LV-YBX-1-ShRNA was purchased from Genechem Co. Ltd (Shanghai, China).

### EdU assay

Purchasing the EdU assay kit from RiboBio (Guangzhou, China) and conducting assay as the manufacturer’s protocol. cells were incubated in EDU solution (Reagent A) diluted with cell medium in a 1000:1 ratio at 37 °C for 2 h, subsequently washed and fixed with PBS and 4% paraformaldehyde, and permeabilized with 0.1% Triton X-100. Protected from light, cells were incubated with TUNEL reaction mixture for 0.5 h. Rinsed with PBS, the samples were detected under a fluorescence microscope. The number of cells was determined by Hoechst staining.

### Transwell assay

Uncoated and Matrigel™ coated Transwell^®^ inserts (Corning) were used to measure invasion. Dilute the BD glue with a ratio of 1:9 and spread the configured BD glue into the upper chamber and incubate at 37 °C for 6 h. Specifically, 700 μl of conditioned medium were added into the lower chamber and 1 × 10^5^ A2780 cells were transferred to each upper chamber. The cells remaining in the upper chamber were removed with a cotton swab after 24 h, fixed, and stained with 0.5% crystal violet. The cells passing through the membrane were counted under a Zeiss Axiovert 200 M light microscope.

### Wound-healing assay

The A2780 cells transfected with indicated constructs plasmids were seeded in a six-well plate with a density of 1.5 × 10^4^ cells/well, maintained in cell incubator overnight. The cell monolayer was scratched with a 200-μl pipette tip as described previously. Cells were washed with PBS and incubated with conditioned medium for 72 h. Images at time points and at matching reference coordinates were taken by immunofluorescence microscopy.

### TUNEL assay

Apoptosis was tested with TUNEL Apoptosis Detection Kit purchased from Beyotime (Shanghai, China) according to the manufacturer’s protocol. Briefly, cells were washed with PBS, fixed in 4% paraformaldehyde, and permeabilized with 0.1% Triton X-100. Then, incubated in TUNEL reaction mixture at 37 °C for 90 min and protected from light. Washed three times with PBS, the samples were detected under a fluorescence microscope (Leica DMI4000B).

### Label-free quantitative identification of ubiquitinated proteins liquid chromatography-tandem mass spectrometry (LC-MS/MS)

After the protein was hydrolyzed by the enzyme, the ubiquitinated peptides were enriched by the motif antibody with high affinity for ubiquitinated lysine (K-GG), and then the ubiquitinated modification sites were analyzed by LC-MS/MS, so as to realize the qualitative and quantitative analysis of large-scale ubiquitinated proteins.

### Confocal microscopy

The A2780 cells were cotransfected with GFP-SIAH1 plasmids (green) and Cherry-YBX-1 plasmids (red) and then co-cultured with MG132 after 36 h. Forty-eight hours after transfection, washing cells with PBS and fixing cells with 4% paraformaldehyde for 0.5 h. Then, staining the cells with DAPI. The labeled SIAH1 and YBX-1 in cells were detected by a confocal microscope.

### Western blotting (WB)

The proteins were extracted in RIPA buffer (Pierce, Rockford, IL, USA) with 1% PMSF and boiled for 8–10 min, and separated by 8% sodium dodecyl sulfate-polyacrylamide gel electrophoresis. Then transferring protein to a PVDF membrane, blocking it with 5% skim milk for 3 h, and incubating with different primary antibodies and secondary antibodies successively. The primary antibodies used in WB included anti-Flag (Cell Signaling #14793), anti-Ub (Santa Cruz #F2819), anti-SIAH1 (Abcam ab2237), anti-YB-1 (Abcam ab12148), anti-GPADH (Cell Signaling D16H11), anti-GFP (Proteintech 66002-1-Ig).

### Cycloheximide (CHX) chase assay

Thirty-six hours after cell transfection with plasmids, 40 mM CHX was added to each well. Preparing cell lysates at the indicated time after CHX treatment and completing WB with indicated antibodies.

### Coimmunoprecipitation assays

After transient transfection GFP labeled plasmids into HEK293T cells, the cells were lysed as described before. The magnetic beads (Beyotime, Shanghai, China) were incubated with the GFP antibody at room temperature for 2 h. The magnetic beads were then incubated with cell extracts (1 mg) at 4 °C for overnight, collecting magnetic beads with a magnetic separation rack. The beads were washed in lysis buffer, mixed with 5× Loading buffer, boiled 25 min, and detected by WB.

### In vitro ubiquitination assay

First, the magnetic beads (Beyotime, Shanghai, China) were incubated with the indicated antibody at room temperature for 2 h, then wash and collect the beads and added into the prepared cell lysate lysed in IP Lysis Buffer (Thermo USA), incubated overnight at 4 °C. The immunoprecipitates were washed 4–5 times with IP Lysis Buffer before being collected by magnetic separation rack (Cell Signaling). Add 60 μl IP Lysis Buffer and 5× SDS-PAGE loading buffer, boil for 25 min and collect samples, immunoblotted with the indicated antibodies. Detecting the proteins with enhanced chemiluminescence (Pierce, Rockford, IL, USA).

### Real-time quantitative PCR and RNA stability assay

All cell RNA extraction and cDNA synthesis were done as previously described. Real-time PCR was performed using SYBR Green Real-Time PCR Master Mix (Vazyme Biotech Co., Ltd, Nanjing, China) and the PCR conditions were set up according to the manufacturer’s protocol. The primers sequence of mRNAs are listed in Supplementary Table S3. The raw RT-qPCR data of mRNAs were normalized to spiked GAPDH. The 2- △△ Cq method was used to analyze and quantify the mRNA-expression levels. For RNA stability assay, actinomycin D was added into cells medium for 0, 3, 6, and 9 h. RNA was extracted and detected as described before.

### Immunohistochemistry

Immunohistochemistry (IHC) staining were conducted as previously described using Goat monoclonal anti- SIAH1 (Abcam, #ab2237, dilutions: 1:250), Rabbit monoclonal anti- YBX-1 (Abcam, #ab12148, dilutions: 1:1000) or Rabbit anti-Ki-67 (Bioworld, #bs1454, dilutions: 1:200). Images were taken using SCN400 Slide Scanner (Leica Microsystems).

### Animal experiment

The 5–6-week-old female nude mice were purchased from (Shanghai SLAC Laboratory Animal Co., Ltd). Mice were randomly divided into six groups with six mice in each group. A2780 cells transfected with the corresponding lentivirus were injected subcutaneously into nude mice. We chose an inoculation concentration of 1 × 10^6^ cells per mouse. The tumor volume and body weight of the mice was measured weekly at the injection site with a caliper when the tumor grew to 2 mm in size on the same day. When more than half of the tumors reached 5 mm, intraperitoneal injection of the antitumor drug DDP (5 mg/kg), The drug was injected twice a week, and the tumor was collected after 3 weeks. Laboratory animal care and procedures were carried out following the animal ethics guidelines of Jiangsu university animal experiment center.

### Statistical analysis

The statistical analysis was completed using SPSS or GraphPad Prism 6. Experiments were performed three times in our study, and results were presented as the means ± SD. The statistical significance of differences between the two groups was assessed by the Student’s unpaired *t* test. One-way ANOVA was used to compare the means among multiple groups. *P* values <0.05 were considered statistically significant.

## Supplementary information

figure S1-S4

supplementary table S1

supplementary table S2

supplementary table S3

## Data Availability

The datasets used and/or analyzed during this study are available from the corresponding author on reasonable request.

## Blinding statement

The researchers were unaware of the group assignment during the experiment and/or when evaluating the results.
