# Supplementary material for: SIAH1 reverses chemoresistance in epithelial ovarian cancer via ubiquitination of YBX-1
Source: Oncogenesis. 2022 Mar 10;11(1):13. doi: 10.1038/s41389-022-00387-6 (PMC8913663; doi:10.1038/s41389-022-00387-6)
Supplement: Supplementary file 1 — figure S1-S4 [file 41389_2022_387_MOESM1_ESM.docx]

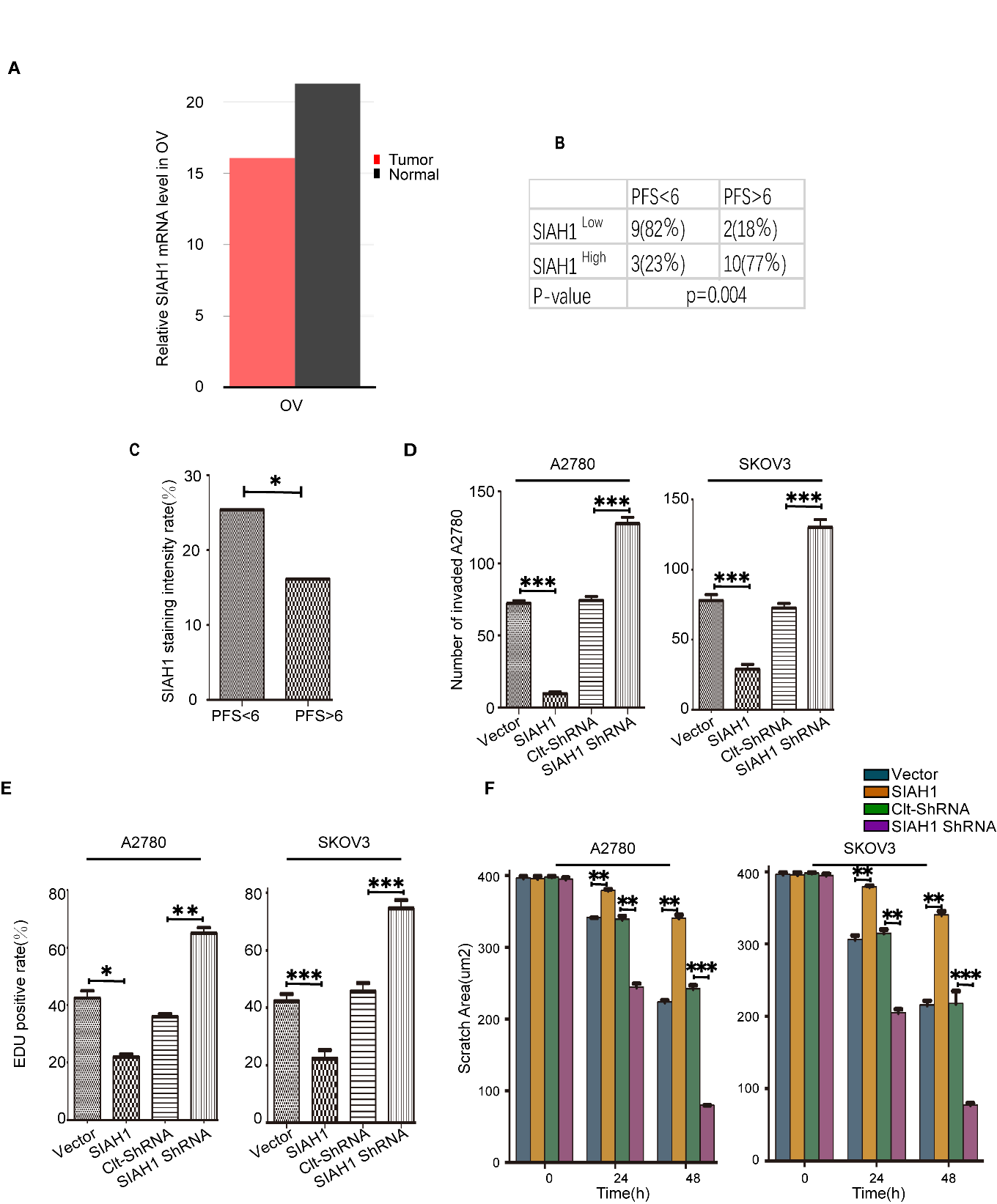


Figure S1. (A)The gene expression profile across all tumor samples and paired normal tissues. The height of bar represents the median expression of certain tumor type or normal tissue. (B) Association between SIAH1 expression with PFS of EOC patients. The staining positive rate is higher in the PFS>6 group than in the PFS>6 group (p < 0.05). (C). The SIAH1 expression in patient tissues of PFS>6 and PFS<6 were assessed and described with statistical graph. Transwell assay (D), EDU assay (E), Wound healing assay (F). Invasion inhibition、Proliferation inhibition、Migration inhibition mediated by overexpressing SIAH1 in A2780 and SKOV3 cells. On the contrary, the invasion、proliferation、migration were promoted by knocking down SIAH1. And the results were presented with statistical graph.


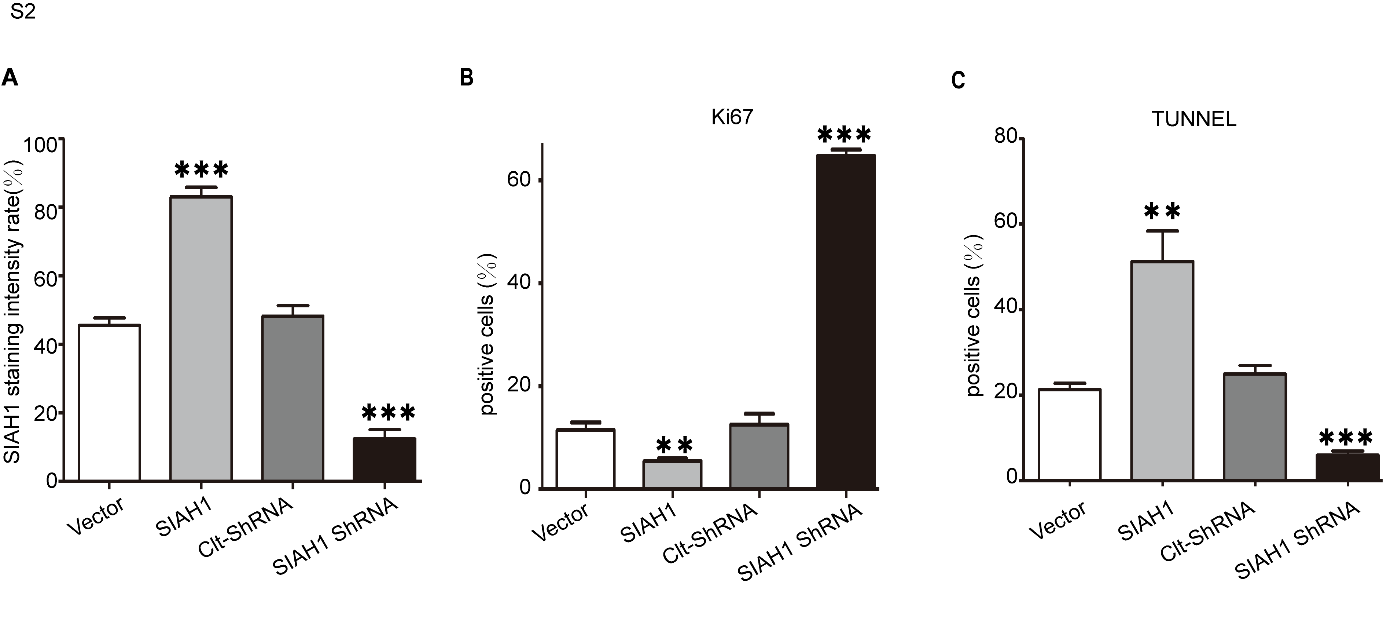


**Figure S2.** (A)The expression of SIAH1 in xenograft tumour tissues overexpressed or knocked down SIAH1 were tested with IHC, and presented with statistical bar chart. (B)、(C) The proliferation and apoptosis of each group xenograft tumour tissues were detected with Ki67 staining and TUNEL assay, and the differences between each group clearly shown in the diagram.


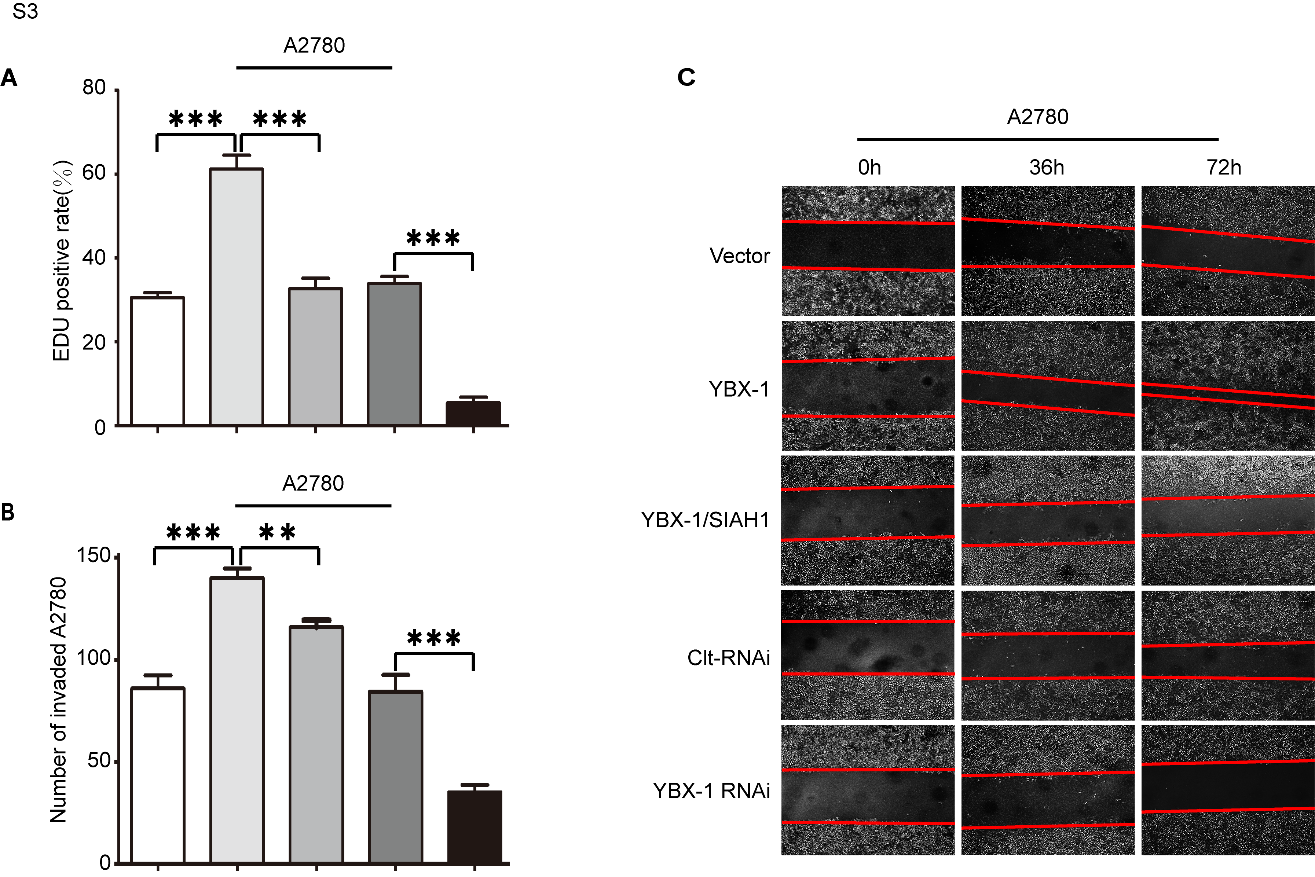


Figure S3. (A) The proliferation、(B) invasion and (C)migration of A2780 cells overexpressing or knocking down YBX-1 were detected by EDU assay、transwell assay and wound healing assay separately. At the same time, the effect of SIAH1-1 on YBX-1 function is also assessed by co-expressing SIAH1 and YBX-1 in A2780 cells. The results were shown with statistical graph.


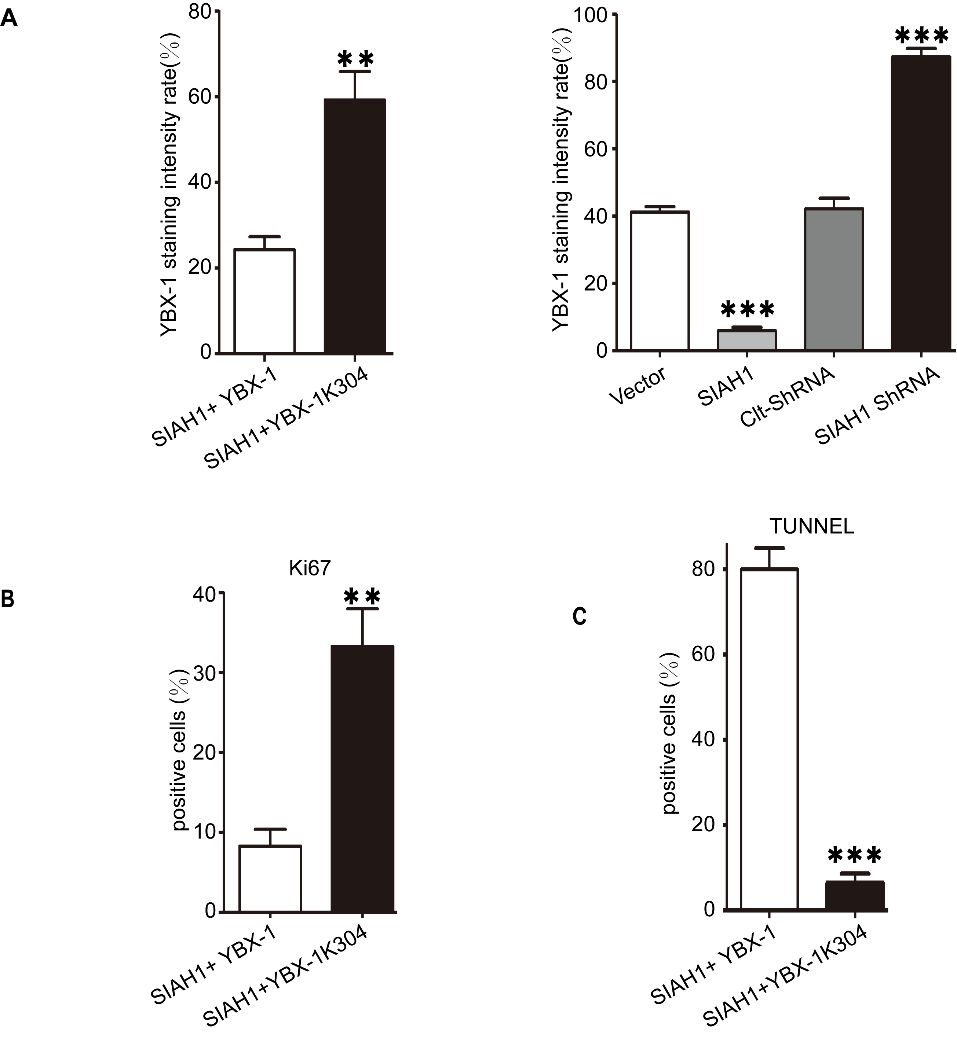


Figure S4. (A)IHC of YBX-1 and SIAH1 expression in tumor tissues of each group and the dates calculated with graphs. (B) KI-67 staining positive rate in tumors treated as indicated were assessed. (C) The apoptosis in tumors treated were detected with TUNEL analysis and presented with graph.
